# Correlations between comorbidities in trials and the community: An individual-level participant data meta-analysis

**DOI:** 10.1177/26335565231213571

**Published:** 2023-11-09

**Authors:** Jamie Crowther, Elaine W Butterly, Laurie J Hannigan, Bruce Guthrie, Sarah H Wild, Frances S Mair, Peter Hanlon, Fergus J Chadwick, David A McAllister

**Affiliations:** 1 School of Health and Wellbeing, College of Medical, Veterinary and Life Sciences, 347493 University of Glasgow, Glasgow, UK; 2 56634 Nic Waals Institute, Lovisenberg Diaconal Hospital, Oslo, Norway; 3 Center for Genetic Epidemiology and Mental Health, Norwegian Institute of Public Health, Oslo, Norway; 4 Population Health Sciences, Bristol Medical School, University of Bristol, Bristol, UK; 5 Usher Institute, College of Medicine and Veterinary Medicine, 172239 University of Edinburgh, Edinburgh, UK; 6 34549 Biomathematics and Statistics Scotland, Edinburgh, UK; 7 School of Biodiversity, One Health and Veterinary Medicine, College of Medical, Veterinary and Life Sciences, University of Glasgow, Glasgow, UK

**Keywords:** Comorbidity, multimorbidity, trials, community

## Abstract

**Background:**

People with comorbidities are under-represented in randomised controlled trials, and it is unknown whether patterns of comorbidity are similar in trials and the community.

**Methods:**

Individual-level participant data were obtained for 83 clinical trials (54,688 participants) for 16 index conditions from two trial repositories: Yale University Open Data Access (YODA) and the Centre for Global Clinical Research Data (Vivli). Community data (860,177 individuals) were extracted from the Secure Anonymised Information Linkage (SAIL) databank for the same index conditions. Comorbidities were defined using concomitant medications. For each index condition, we estimated correlations between comorbidities separately in trials and community data. For the six commonest comorbidities we estimated all pairwise correlations using Bayesian multivariate probit models, conditioning on age and sex. Correlation estimates from trials with the same index condition were combined into a single estimate. We then compared the trial and community estimates for each index condition.

**Results:**

Despite a higher prevalence of comorbidities in the community than in trials, the correlations between comorbidities were mostly similar in both settings. On comparing correlations between the community and trials, 21% of correlations were stronger in the community, 10% were stronger in the trials and 68% were similar in both. In the community, 5% of correlations were negative, 21% were null, 56% were weakly positive and 18% were strongly positive. Equivalent results for the trials were 11%, 33%, 45% and 10% respectively.

**Conclusions:**

Comorbidity correlations are generally similar in both the trials and community, providing some evidence for the reporting of comorbidity-specific findings from clinical trials.

## Background

Multimorbidity, the presence of two or more long-term conditions, is a common and increasing problem that creates challenges for patients, clinicians and guideline developers.^1,2^ One such challenge is the fact that comorbidity (the presence of other diseases in addition to an index condition and a necessary consequence of multimorbidity) is under-represented in randomised controlled trials. Since trials inform treatment recommendations,^3^ this leads to uncertainty about the applicability of trial findings to people with multimorbidity.

We previously compared comorbidity counts, and the prevalence of specific comorbidities, among trial participants and people in the community with the same index condition; we found that the average comorbidity count (based on 21 comorbidities) in trials was around half of the average in the community.^3^ This meant that comorbidity was under-represented rather than absent from trials, suggesting that trialists could analyse and report treatment effects for people with multimorbidity, helping inform treatment decision making.

Such reporting is not straightforward, however, as there are many potential combinations of conditions, and it is not feasible to report treatment effects for all combinations. To help inform reporting, it is therefore useful to understand patterns of comorbidities in trials, and to determine whether patterns are similar in trials and the community. We expect some correlations to be stronger in the community than in trials due to selection bias against recruiting multimorbid patients in clinical trials.

Previous studies have examined patterns of multimorbidity in the community,^4–6^ mostly using clustering and network-based approaches. However, we are not aware of any study which has examined patterns in trials, nor of any study that has compared comorbidity patterns in trials and the community. Therefore, to study patterns of comorbidity, we compared correlations between commonly occurring comorbidities in clinical trials and the community.

## Methods

### Study design

This study uses Individual-level Participant Data (IPD) to estimate correlations between comorbid conditions (comorbidities) in trial participants and compares these to correlations between comorbidities in people in the community with the same index condition (defined below) identified from electronic-health records. Correlations between comorbidities were estimated using Bayesian multivariate probit models conditioning on age and sex for each trial and for the community. Using 3,000 samples from each model, estimates from each trial were combined into a single weighted average for each index condition (based on the number of participants), which was then compared to the community estimates.

### Data sources

We identified IPD from industry sponsored drug trials from two repositories and a community sample from a large database of routine healthcare data from the UK. For a detailed description see Hanlon et al., 2019.^3^ Briefly, trials were selected based on a predefined protocol (Prospero CRD42018048202). Individual-level participant data were accessed from 83 trials (54,688 participants) from Yale University Open Data Access (YODA) and the Centre for Global Clinical Research Data (Vivli) for 16 index conditions (Table 1). Due to changes in the repositories holding the trial data there are fewer trials, hence, fewer index conditions than in our previous study.^3^ Community patient data were obtained from the Secure Anonymised Information Linkage (SAIL) databank for all individuals (860,177) with one of the 16 index conditions identified in the trials. SAIL is a safe haven, holding general practice medical records for 70% of the Welsh public^7^ of whom it is representative with respect to age, sex and socio-economic status.^3^

**Table 1.** Community – Trial comparison summary.

Condition	Community N	Trial N	N trials	Community mean age	Trial mean age	Community age SD	Trial age SD	Community male %	Trial male %
Asthma	169912	1627	4	46	42	17	16	43.7	40.1
Axial spondyloarthritis	1865	352	1	51	40	14	12	76.7	71.3
Benign prostatic hyperplasia	18902	2816	6	71	64	9	8	100	100
Dementia any	11357	5811	6	79	74	7	8	35.5	40.7
Diabetes mellitus type 2	79789	17885	22	64	57	12	10	56.6	52.9
Hypertension	292736	6209	10	65	56	12	10	48.6	58.3
Osteoarthritis	118577	1320	1	66	64	11	12	40.8	32.7
Osteoporosis	34295	2055	2	71	61	11	12	11	4.1
Parkinson disease	4236	1761	4	73	63	9	10	62.2	58.9
Psoriasis	46578	771	2	48	48	15	12	47.1	63.4
Psoriatic arthritis	3400	822	3	53	47	13	11	48	55
Pulmonary disease chronic obstructive	54323	4380	6	68	64	10	8	51.6	65.5
Pulmonary hypertension	594	406	1	69	55	14	16	38.2	21.7
Restless legs syndrome	9961	2065	5	60	54	13	12	28.2	37.9
Rheumatoid arthritis	12729	4715	8	60	52	14	12	29.2	20.4
Systemic lupus erythematosus	923	1693	2	50	38	13	11	10.1	6

SD – Standard Deviation. N – Number.

### Index conditions

In the trials, index conditions were classified based on the indication recorded in the trial registration. We identified the same index conditions in the community using codes from the Read classification system, which is the standard coding system used within primary care in the UK.^8^ We extracted data from all individuals in SAIL with any one of the 16 index conditions identified in the trials (asthma, axial spondyloarthritis, benign prostatic hyperplasia, dementia (any), diabetes mellitus type 2, hypertension, osteoarthritis, osteoporosis, Parkinson disease, psoriasis, psoriatic arthritis, pulmonary disease chronic obstructive, pulmonary hypertension, restless legs syndrome, rheumatoid arthritis and systemic lupus erythematosus).

### Identification of comorbidities

In both the trials and community, comorbidities were defined as any of 21 pre-specified conditions (cardiovascular disease, chronic pain, arthritis, affective disorders, acid- related disorders, asthma/chronic obstructive pulmonary disease, diabetes mellitus, osteoporosis, thyroid disease, thromboembolic disease, inflammatory conditions, benign prostatic hyperplasia, gout, glaucoma, urinary incontinence, erectile dysfunction, psychotic disorders, epilepsy, migraine, parkinsonism and dementia),^3^ present in addition to the trial index condition. As previously described,^3^ it was not possible to consistently identify comorbidities using trial participant medical history as this was often redacted to reduce the risk of individuals being re-identified, moreover medical history data is collected very differently in the trial and community data (clinical research forms and coding for clinical care respectively). Therefore, we defined comorbidities in both datasets using patient concomitant medication to allow comparison between trial participants and the community. Patient concomitant medication was classified using the World Health Organization Anatomic Therapeutic Chemical (WHO-ATC) system.^9^ This allowed identical definitions to be applied to both the trials and the routine data. We determined concomitant medications as any drug started before or on the trial randomisation date. In the community, the NHS Business Authority ATC to Read code lookup table was used,^10^ and for drugs not included in the look up table we matched Read code-defined drugs to ATC codes. In total we identified 21 comorbidities using concomitant medication. To reduce the dimensions and complexity of the comparisons, we then identified the six most prevalent comorbidities for each index condition in the community, and examined the same six comorbidities in the trials for each index condition (regardless if these six comorbidities were not the most common in the trials for that index condition).^3^

### Statistical analysis

Summary statistics for age, sex and the prevalence of each comorbidity were calculated for all trial participants and individuals in the community. Correlations between comorbidities were estimated using a Bayesian multivariate probit model,^11^ as implemented in Chadwick et al., 2022.^12^ We ran one model per trial, and one model per index condition in the community. All pairwise correlations were simultaneously estimated, conditional on age and sex. This type of model is well suited to the binary correlated nature of our data, where comorbidities are expected to be associated, and the prevalence of each comorbidity is likely to be related to age^13^ and sex. We chose this approach, rather than clustering or network-based analyses, as it provides quantitative estimates of relations between comorbidities which can then be compared between trials, and between trials and the community.

### Model structure

The multivariate probit model^11^ and a detailed account of the modelling can be found in Additional file 1 in the Supplemental Material. Multivariate models allow multiple correlated response variables, in this case the presence or absence of the six most common comorbidities for that index condition. The response variables are linked to a linear predictor by means of a probit link function. The linear predictor consisted of the intercept, age (in years) and sex (male versus female). The response variables are allowed to correlate with each other by means of a correlation matrix (which is conditioned on age and sex through the linear predictor). Using a Bayesian implementation of the multivariate probit allowed us to estimate full posteriors, both quantifying and allowing propagation of the uncertainty in the correlation estimates.

### Prior choice and model convergence

We fitted all models using minimally informative priors. For the intercept, age and sex coefficients, we used standard normal priors (a mean of 0 and variance of 1). For the correlation prior we used the Lewandowski-Kurowicka-Joe (LKJ) distribution.^14^ The LKJ has a single parameter which determines the amount of shrinkage on the marginal correlations. We chose a value designed to give minimal shrinkage. Three independent Markov Chain Monte Carlo (MCMC) were initiated per model, each giving 1000 samples (MCMC draws) per model. Sampling diagnostics and MCMC convergence were checked (rhat < 1.05, divergent transitions < 2% and Effective Sample Size > 100 times the number of MCMC chains) for all correlation parameters in all models. We adjusted model fitting parameters where diagnostic tests indicated poor sampling or convergence.

### Model implementation

For the community, we used aggregate data extracted from the SAIL safe haven to simulate pseudo-IPD (sampling from a truncated normal distribution bounded at zero for each combination of comorbidities) for each index condition. See Model implementation in Additional file 1 in the Supplemental Material for further details. We restricted the minimum and maximum ages of the simulated community IPD to match that of the trials for each index condition. Models were fitted to both the restricted and non-restricted IPD and compared with the same index condition in the trials in a sensitivity analysis; we present the results from the age restricted models. For index conditions with more than 20,000 patients we obtained a random sample of 20,000 individuals to reduce computation time.

For each trial we fitted a model within the relevant secure environment (YODA or Vivli). We then exported samples from the posterior (3,000 MCMC draws). The sample estimates from trials which shared the same index condition were combined into a single weighted average for each index condition (based on the number of participants). This weighted trial estimate was then compared with the community estimate for the same index condition. Since nearly all the correlations were positive, we compared correlations by simple subtraction (age restricted community estimate - weighted trials estimate) for each sample. For the trials, community and differences, the samples from the posterior distribution were summarised via the mean to obtain a central estimate, and via the 2.5^th^ centile and 97.5^th^ centile to obtain a 95% credible interval.

All data processing was done in R,^15^ and models were fitted using a variant of Hamiltonian Monte Carlo in the Stan programming language^16^ using the Rstan^17^ and cmdstanr^18^ packages. Code for the analysis can be found on GitHub.^19^

## Results

Table 1 shows summary statistics for the trials and community for each index condition. Across index conditions, the median number of individuals in the community was 15,816, and ranged from pulmonary hypertension with fewer than 600 individuals to hypertension with nearly 300,000. The median number of trials per index condition was four and ranged from 1 to 22. The number of trial participants (median: 1908) ranged from 352 for axial spondyloarthritis to nearly 18,000 for type 2 diabetes. For the same index condition, individuals in the community had the same or higher mean age than in the trials (Table 1). The median age of individuals in the community and trials was 65 and 56 respectively. The variation in age (standard deviation) was similar across all index conditions for both the trials (median: 11.5) and community (median: 12.5).

**Figure 1.**
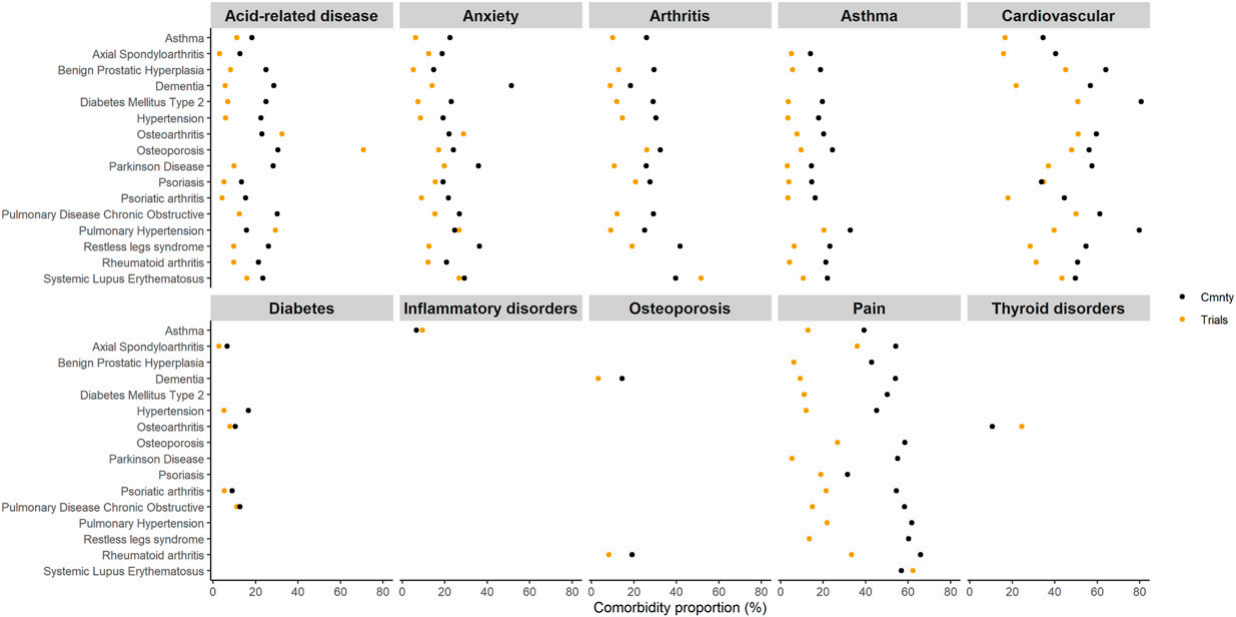
Proportion of individuals with the six most common comorbidities for each of the 16 index conditions compared between the community and trials. Ten comorbidities comprise the six most common comorbidities (based on community prevalence) across all index conditions. Black indicates the community and orange the trials.

As previously reported,^3^ most comorbidities were more common in the community than in trials (Figure 1). Exceptions included acid-related diseases for the index conditions osteoarthritis, osteoporosis and pulmonary hypertension; anxiety for individuals with osteoarthritis and pulmonary hypertension; and inflammatory disorders in individuals with asthma. Of the 21 defined comorbidities only ten were among the six commonest for one or more of the 16 index conditions: acid-related disease, anxiety, arthritis, asthma, cardiovascular disease, diabetes mellitus, inflammatory disorders, osteoporosis, pain and thyroid disorders. For eight index conditions, the same six comorbidities were commonest: acid-related disease, anxiety, arthritis, asthma, cardiovascular disease and pain.

**Figure 2.**
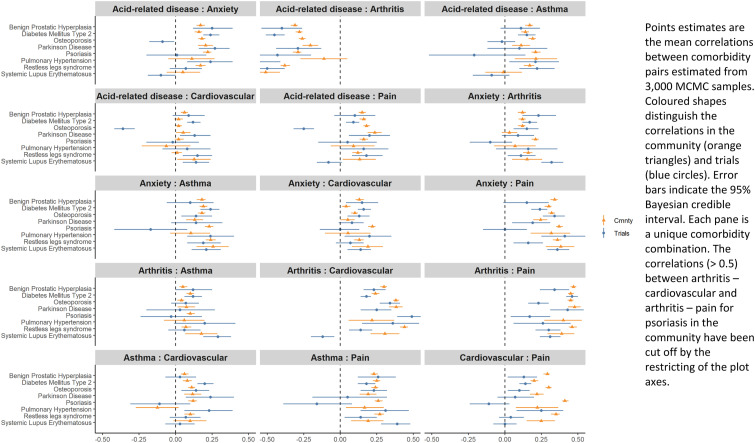
Estimated pairwise correlations between the six most common comorbidities in the community and trials for the eight index conditions which share the same six most common comorbidities.

**Figure 3.**
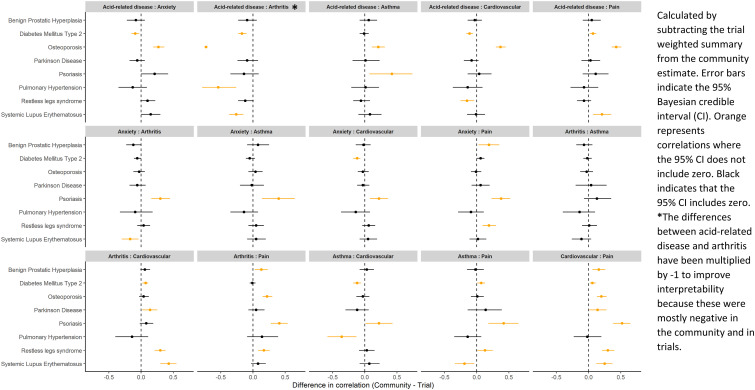
Differences between estimated correlations in the community and trials for the eight index conditions which all share the same six most common comorbidities.

In the community, 5% of correlations were negative (<= -0.1), 21% were null (-0.09 to 0.09), 56% were weakly positive (0.1 to 0.29) and 18% were strongly positive (>= 0.3) (Table 2). Equivalent results for the trials were 11%, 33%, 45% and 10% respectively (Table 2). The median (25^th^ percentile, 75^th^ percentile) correlation in the community was 0.16 (0.09, 0.23) compared with 0.12 (0.01, 0.2) in the trials (Table 2). Where observed correlations are likely artefacts of our concomitant medication based definitions (e.g. we only used antiacid medications to identify individuals with acid-related diseases if those individuals were not also taking non-steroidal anti-inflammatory drugs, which would be expected to induce a negative correlation between arthritis and acid-related disease) these are presented in the figures but are not discussed in the text. To simplify the presentation of our results we focus on the eight index conditions (benign prostatic hyperplasia, diabetes mellitus type 2, osteoporosis, Parkinson disease, psoriasis, pulmonary hypertension, restless legs syndrome and systemic lupus erythematosus) which share the same six most common comorbidities (based on community prevalence) in Figure 2 and Figure 3. Results for the remaining index conditions are presented in Additional file 2 and Additional file 3. The direction and magnitude of correlations were generally similar across index conditions for the same comorbidity pair (Figure 2). For trials, a similar pattern was mostly seen, but with wider 95% Credible Intervals (CI). The exceptions to this were systemic lupus erythematosus trials (two trials) in which the arthritis-cardiovascular pair was negatively correlated, and the single osteoporosis trial which had several negative correlations.

**Table 2.** Summary of point estimates for the 16 index conditions.

	Median correlation (25^th^, 75^th^ percentiles)	Number of comorbidity pairs	Number (%) of comorbidity pairs			
			Negative (r <= -0.1)	Null (-0.1 < r < 0.1)	Weakly positive (0.1 <= r < 0.3)	Strongly positive (r >= 0.3)
Community	0.16 (0.09, 0.23)	240	13 (5.4%)	50 (20.8%)	134 (55.8%)	43 (17.9%)
Trials	0.12 (0.01, 0.2)	240	27 (11.2%)	79 (32.9%)	109 (45.4%)	25 (10.4%)

r = Correlation coefficient.

On comparing correlations between the community and trials, 21% of correlations were stronger in the community, 10% were stronger in the trials and 68% were similar in both. For most cases where differences in the correlation were evident, such as most of the comorbidity pairs in psoriasis (Figure 3), this was because correlations were positive in the community, but were null (-0.09 to 0.09) or the 95% CI included zero for the trials (Figure 2). Similar results for the correlations, and differences between the correlations in trials and the community, were evident for the remaining eight index conditions which included other comorbidity pairs (Additional file 2 and Additional file 3). In the sensitivity analysis where we compared trials and the community without restricting the community to the age-range seen in the trials, we observed similar results to those found in the main analysis.^19^

## Discussion

Using individual-level patient data, we compared the patterns of comorbidity for 16 index conditions in over 850 thousand individuals in the community and over 50 thousand trial participants. In the community, for most index conditions, more than two thirds of comorbidity pairs were positively correlated. For the same index conditions and comorbidity pairs, correlations were broadly similar in both trials and the community. Thus, while comorbidity is more prevalent in the community than trials, among those with comorbidity, the pattern of correlations were mostly similar.

Although a number of studies have examined patterns of comorbidity in the community,^4–6^ or looked at excluded comorbid conditions in trials.^20^ This is the first study of which we are aware to examine patterns of comorbidity in trials, or to compare comorbidity patterns between trials and the community. The study benefited from a large amount of trial and community derived IPD. Nonetheless, there are a number of limitations. First, comorbidities were identified using patient concomitant medications. This meant that not all conditions of interest could be studied because they are not associated with specific drugs. Some conditions had to be collapsed into very broad categories (e.g. cardiovascular disease as a single category). In addition, inflammatory and autoimmune conditions could not be distinguished therefore they were classed together as inflammatory. Also, some misclassification is likely to have occured. Secondly, we were unable to measure the severity of specific comorbid conditions, and severity may be worse, on average, in the community than in trials. More importantly for the purposes of studying relationships between comorbidities, our concomitant medication based approach meant that some correlations were present almost by definition (e.g. we did not use antacid drugs to define acid related diseases in people taking non-steroidal drugs inducing a negative correlation). Nonetheless, this approach was based on pre-specified definitions and allowed us to consistently estimate correlations in both trials and the community in a way which is unlikely to have been affected by these limitations differentially.

Previous studies examining patterns of comorbidity in the community have mostly used statistical dimension reduction techniques to derive clusters.^4,21,22^ Cluster-based methods aim to simpify the high-dimensional problem posed by multimorbidity (e.g. for six comorbidities there are 64 combinations) by assigning individuals to a manageable number groups based on their within-group similarity. In contrast, we used multivariate models as these allowed us to estimate correlations between comorbidities condition on age and sex, and to compare these between trial and community settings. While this approach, like clustering, makes a number of assumptions (e.g. the correlations between comorbidities are constant across levels of the covariates), it does allow comparison of correlations between settings in a way which accounts for uncertainty in the trial/community based estimates.

Notwithstanding the choice of methods for summarising comorbidities (e.g. selected conditions, simple counts, or clustering approaches), our finding that comorbidity correlations are mostly similar in trials and the community is a potentially useful observation. A key feature of multimorbidity (and of comorbidity in trials) is the presence of disease-disease interactions, disease-drug interactions, and drug-drug interactions. Each of these depends on relationships between specific comorbidities (e.g. where treatment of one condition, such as antiplatelets for coronary artery disease, adversely impacts another condition, such as peptic ulcer disease). Clinical trial data is underutilised in assessing the impact of multimorbidity, as it is often hampered by a lack of reporting of comorbidity specific findings. One reason for this lack of reporting may be uncertainty as to whether clinical trial participants with comorbidity are similar to patients with comorbidity in community settings. As such, the observation that correlations are similar in trials and in the community is important. It provides some evidence that findings from trials concerning individuals with specific comorbidities may be relevant to many individuals with the same comorbidities in the community. International standards for the reporting of comorbidity and multimorbidity trials are needed, informed by clinical expertise and an understanding of the possible effects of concordant and discordant conditions. Our results suggest that patterns of multimorbidity identified in routine health care data, which is much richer in people with multimorbidity, may also help inform the development of such standards.

We found that correlations between common comorbidities were similar in the community and trials. This is potentially an important observation for comorbidity and multimorbidity research, as it suggests that results for relations between comorbidities may be extrapolated between settings. However, to have confidence in doing so, it would be important to first demonstrate that similar correlations are also found across different population characteristics (e.g. different countries, different socio-economic groups) and for a disease-measure not based on medications.

## Conclusions

For most index conditions, despite differences in the prevalence of individual comorbidities, pairs of comorbidities were mostly similar in trials and the community. This suggests that while multimorbidity is under-represented in trials, patterns of comorbidity may be similar in both settings. This provides some support for the reporting of comorbidity-specific findings from clinical trials.

## Supplemental Material

Supplemental Material - Correlations between comorbidities in trials and the community: An individual-level participant data meta-analysis Click here for additional data file. Supplemental Material for Correlations between comorbidities in trials and the community: An individual-level participant data meta-analysis by Jamie Crowther, Elaine W Butterly, Laurie J Hannigan, Bruce Guthrie, Sarah H Wild, Frances S Mair, Peter Hanlon, Fergus J Chadwick, and David A McAllister in Journal of Multimorbidity and Comorbidity

## Appendix

### Abbreviations

ATC -: Anatomic Therapeutic Chemical
CI -: Credible Interval
ESS -: Effective Sample Size
IPD -: Individual-level Participant Data
LKJ -: Lewandowski-Kurowicka-Joe
MCMC -: Markov Chain Monte Carlo
MVP -: Multivariate Probit
SAIL -: Secure Anonymised Information Linkage
YODA -: Yale University Open Data Access
