# Supplementary material for: Correlations between comorbidities in trials and the community: An individual-level participant data meta-analysis
Source: J Multimorb Comorb. 2023 Nov 9;13:26335565231213571. doi: 10.1177/26335565231213571 (PMC10637135; doi:10.1177/26335565231213571)
Supplement: Supplemental Material - Correlations between comorbidities in trials and the community: An individual-level participant data meta-analysis [file sj-pdf-1-cob-10.1177_26335565231213571.pdf]

Supplementary Methods

The following text outlines the statistical methods in more detail, the code for the analysis can be found on GitHub (1).

Statistical analysis

All data wrangling and analyses were done in R (2), and models were fitted using a variant of Hamiltonian Monte Carlo in the STAN programming language (3) using the Rstan (4) and cmdstanr (5) packages.

Model structure

The multivariate probit model used in this study, as defined by Albert & Chib, 1993 (6):

Probit link function

$$y_{id} = I(z_{id} > 0)$$

Linear predictor

$$z_i = x_i\beta + \epsilon_i$$

$$z_{id} = \sum_{j=1}^{J} x_{ij}\beta_{jd} + \epsilon_{id}$$

Error term

$$\epsilon_i \sim N(0, \Omega)$$

$$\Omega_{ii} = 1$$

Priors

$$\beta \sim N(0, 1)$$

$$\Omega \sim LKJ(1)$$

The multivariate probit model takes the presence or absence of comorbidities as binary outcomes (y_{id}) where i is the ith of N patients and d is the dth of D diseases, and i runs from 1 to N and d runs from 1 to D giving a single value (y_{id}). The probit function (I) links these binary outcomes to the continuous latent state (z_{id}). An outcome of zero corresponds to a negative latent state, and an outcome of one corresponds to a positive latent state. The vector of continuous latent variables

($\boldsymbol{z}_i$) are the sum of the linear predictor ($X_i\beta$) and an error term (ϵ_i) assumed to come from a multivariate normal distribution. This allows us to condition the correlations (Ω) on J risk factors, in this case age and sex. $\boldsymbol{X}$ is an N x J design matrix with columns corresponding to the covariates (in our case, the intercept (= 1), age (in years) and sex (0 or 1) and one row per patient. The coefficient matrix, $\boldsymbol{\beta}$, defines the relationship between the covariates and the corresponding disease's prevalence. The correlation matrix (Ω) defines the correlations between all the comorbidities.

Prior choice and Model convergence

We fitted all models using minimally informative priors due to lack of prior knowledge of correlations between comorbidities and to minimise bias. This also helps to align our results with equivalent frequentist methods. For the intercept, age and sex covariate coefficients ($\boldsymbol{\beta}$), we used standard normal priors (mean = 0 and standard deviation = 1) which are relatively flat under probit transformation. The Lewandowski-Kurowicka-Joe (LKJ) distribution was used as the prior for the correlations (Ω), this is a prior for correlation matrices and has a single shape parameter eta (η), which we set to one (7). This gives as uniform a probability density as is possible with the LKJ distribution (3). Uniformity is a function of the shape parameter and the dimensions of the matrix, we set η to one giving minimal shrinkage towards zero. Increasing the dimensions of the correlation matrix decreases the average correlation, such that the correlations shrink towards zero. However, for six dimensions there is a low amount of shrinkage.

Three independent Markov Chain Monte Carlo (MCMC) per model were initiated from random initial values and each ran for 1000 warm-up and 1000 sampling iterations. To assess MCMC convergence, we examined the split rhat test (8) for each correlation parameter estimate. Values greater than 1.05 were examined further by visually checking MCMC traceplots for each parameter. Given the complexity of the models and the constrained nature of correlation matrices we expected a small number of divergent transitions, hence, we only examined models further where more than 2% of all iterations for a single MCMC chain resulted in divergent transitions. There were no divergent

transitions in any of the community models. However, in the trials three models had greater than 2% divergent transitions, these models were re-run with higher adapt delta and max treedepth values until the number of divergent transitions were below the 2% threshold. We also examined the bulk and tail Effective Sample Size (ESS) for each correlation parameter. The Stan Development Team recommend that these values are greater than 100 times the number of MCMC chains. In the trials, one model with low bulk and tail ESS was re-ran with a higher number of sampling iterations and another model had bulk and tail ESS less than 300 but greater than 200 and all other sampling diagnostics were met. All parameters in the community models had bulk and tail ESS values above 300. Despite restricting the maximum number of individuals to 20,000 for index conditions in the community, the average model run time was 12 hours. Before this, some models took as long as 179 hours to run even on a high-performance computer with 64GB of ram and 16 cores.

Meta-analysis weighting

We fitted one model per trial, and for trials which shared the same index condition we combined the model results into a single weighted estimate. To do this the MCMC iterations (3 chains per model, 1000 iterations per chain) from all trial models were exported from YODA and Vivli and combined into a single data frame. The estimates from each iteration were multiplied by the number of participants in that trial and divided by the total number of trial participants with that index condition. We then summed these estimates for each MCMC iteration, giving 3000 weighted estimates per index condition. The average correlation between each comorbidity was then calculated, resulting in a single weighted estimate for each comorbidity correlation in each index condition, which could then be compared with the same index condition in the community. The code used to do this is provided in the script titled “32-Prepare_trial_meta_analysis_files” in the scripts folder of the public GitHub repository (1).

Model implementation

For the community data, five index conditions had fewer than 4500 individuals (axial spondyloarthritis, Parkinson disease, psoriatic arthritis, pulmonary hypertension and systemic lupus erythematosus). This meant that for some combinations of comorbidities there were only a small number of individuals. For these index conditions, models were fitted inside the SAIL safe haven. For the remaining eleven community index conditions the models were run on a high-performance computing environment within our institution. For these commoner conditions we aggregated the data within the SAIL repository by stratifying individuals based on sex and each unique combination of comorbidities then counting the number of participants and summarising the age distribution within each stratum (1). Within each stratum, age was approximately normally distributed, so could be summarised with a mean and standard deviation. On fitting the models, we first used these aggregated data to simulate pseudo-IPD (sampling from a truncated normal distribution bounded at zero for each combination of comorbidities) then fitted the models as for IPD. For the subset of five index conditions with fewer than 4500 individuals, we compared the parameter estimates from models fitted using IPD and pseudo-IPD; both methods gave very similar results (1). We restricted the minimum and maximum ages of the simulated community IPD to match that of the trials. Models were fitted to both the restricted and non-restricted IPD and compared with the trials in a sensitivity analysis; we present the results from the age restricted models. For index conditions with more than 20,000 patients we obtained a random sample of 20,000 individuals to reduce computation time.

For each trial we fitted the models within the relevant secure environments (YODA or Vivli). We then exported samples from the posterior (3,000 MCMC draws). The sample estimates from trials which shared the same index condition were combined into a single weighted average for each index condition (based on the number of participants). This weighted trial estimate was then compared with the community estimate for the same index condition. Since nearly all the correlations were positive, we compared correlations by simple subtraction (age restricted community estimate -

weighted trials estimate) for each sample. For the trials, community and differences, the samples from the posterior distribution were summarised via the mean to obtain a central estimate, and via the 2.5th centile and 97.5th centile to obtain a 95% credible interval.

References

1. Crowther J, McAllister DA. *comorbiditiy_correlation_public*. GitHub repository. 2023. https://doi.org/10.5281/zenodo.7829836.

2. R Core Team. *R: A language and environment for statistical computing.* R Foundation for Statistical Computing, Vienna, Austria. 2022. https://www.R-project.org/.

3. Stan Development Team. *Stan User's Guide*. 2023. https://mc-stan.org/docs/stan-users-guide/index.html.

4. Stan Development Team. *RStan: the R interface to Stan*. 2022. https://mc-stan.org/rstan/articles/rstan.html.

5. Gabry J, Češnovar R. *R Interface to CmdStan*. 2022. https://mc-stan.org/cmdstanr/index.html.

6. Albert JH, Chib S. Bayesian Analysis of Binary and Polychotomous Response Data. *Journal of the American Statistical Association*. 1993;88(422):669–79.

7. Lewandowski D, Kurowicka D, Joe H. Generating random correlation matrices based on vines and extended onion method. *J Multivar Anal*. 2009;100(9):1989–2001.

8. Vehtari A, Gelman A, Simpson D, Carpenter B, Bürkner PC. Rank-normalization, folding, and localization: An improved R-hat for assessing convergence of MCMC. *Bayesian Anal*. 2019;16(2):667–718.
